# Optimizing the osteogenic and angiogenic properties of nano-bioactive glass through adjustment of zinc and magnesium ion doping ratios: an *in vitro* study

**DOI:** 10.3389/fbioe.2026.1831550

**Published:** 2026-04-24

**Authors:** Shalitanati Wuermanbieke, Ji Chen, Luhui Zhang, Chengwei Wang

**Affiliations:** 1 The Third Affiliated Hospital of Xinjiang Medical University, Xinjiang Medical University, Urumqi, China; 2 Department of Joint Surgery, Affiliated Hospital of Zunyi Medical University, Zunyi City, China; 3 Guangdong Provincial Engineering Technology Development Center for Packaging Printing Processes and Materials, Guangzhou Vocational University of Science and Technology, Guangzhou, Guangdong, China

**Keywords:** angiogenesis, bioactive glass, magnesium ions, mineralization, osteogenesis, zinc ions

## Abstract

Bioactive glass (BG) is an ideal bone substitute material. Current research focuses on optimizing its performance by introducing bioactive ions and leveraging its degradability for sustained ion release. However, previous studies have mainly concentrated on single-ion doping, neglecting the impact of multi-ion co-doping on glass structure and dynamic release behavior, thereby failing to effectively enhance performance of BG through multi-ion doping strategies. In this study, Zn^2+^ and Mg^2+^ were co-doped into BG at different ratios (Zn/Mg: 0%/20%, 5%/15%, 10%/10%, 15%/5%, 20%/0%) using the emulsion method. The effects of the Zn/Mg ratio on the mineralization behavior, osteogenesis, and angiogenesis of BG were systematically investigated. Results indicated that the 5%Zn/15%Mg group could maintain the amorphous state of the BG for an extended period, inhibiting the formation of hydroxyapatite and thus maintaining a higher ion release concentration. Notably, this specific composition demonstrated optimal osteogenic and angiogenic properties. The study demonstrates that optimizing the multi-ion doping ratio can modulate the mineralization process of BG via synergistic effects and enhance its biological functions, providing a new strategy for designing high-performance bone repair materials.

## 1 Introduction

Bone defects caused by trauma, cancers, congenital anomalies, and other factors significantly impact patients’ quality of life. Despite the fact that autologous, allogeneic, and xenograft bone grafts are routinely employed for bone repair, these biological implants may still limited by donor scarcity, risk of infection transmission, and immune rejection (Mebarki et al., 2017).

**SCHEME 1:**
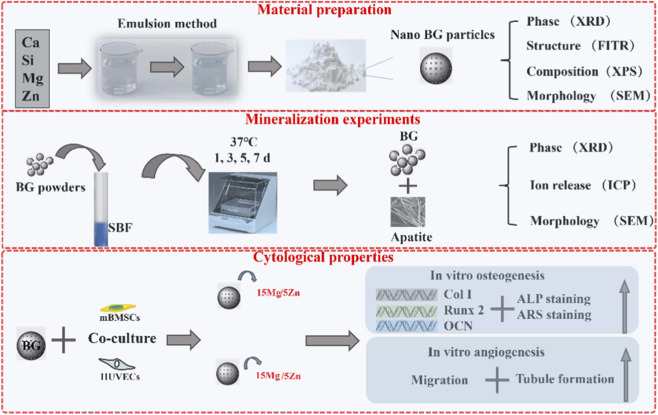
Schematic illustration of the experimental workflow for synthesizing and characterizing Mg/Zn co-doped bioactive glass (BG). rBMSCs: rat bone marrow mesenchymal stem cells; HUVECs: human umbilical vein endothelial cells.

Due to its excellent apatite formation ability, BG (Bioactive glass) has emerged as a promising biomaterial in the field of bone tissue regeneration (Jones et al., 2006). In addition, BG can rapidly degrade while releasing large amounts of bioactive ions, which have been shown to promote osteogenic differentiation and proliferation of cells (Hoppe et al., 2011). Given these properties, significant research has focused on modifying BG through ion doping. Various functional ions have been doped into BG, and their corresponding biological functions have been extensively validated.

Magnesium and zinc are essential trace elements in the human body, playing crucial roles in bone metabolism and development; consequently, they have become primary target ions for BG doping (Zamani et al., 2019; Hohenbild et al., 2021). First, zinc ions can stimulate ATPase activity within cells and protein synthesis in osteoblasts, thereby promoting bone regeneration (Yamaguchi, 1998). Moreover, zinc ions can promote bone regeneration by inhibiting osteoclast gene expression while promoting the expression of a series of osteoblast genes, such as collagen I, alkaline phosphatase (ALP), osteopontin and osteocalcin (Yamaguchi, 1998; Kwun et al., 2010). Specifically, magnesium is the fourth most abundant cation in the human body, with approximately 60% of total body magnesium stored in bone tissue, where it stimulates stem cell proliferation and differentiation, controls apoptosis, and improves the mechanical properties of newly formed bone (Kargozar et al., 2022). Magnesium ions increase cell proliferation and differentiation in a concentration-dependent manner. For instance, it was reported that magnesium ions at concentrations ranging from 2 to 10 mM boosted cell proliferation and differentiation (Nourisa et al., 2021). Furthermore, most studies indicate that ion concentration is a key factor influencing its biological properties (Hoppe et al., 2011; Zamani et al., 2019; Zhang et al., 2024). Additionally, co-doping strategies combining Zn and Mg have shown synergistic effects in enhancing scaffold mechanical properties, antibacterial efficacy, and bioactivity compared to single-ion doping (Zamani et al., 2019). Therefore, this study selected these two ions to investigate their synergistic potential through precise ratio optimization.

The ion concentration released by BG is closely related to its mineralization behavior. The high bioactivity of BG stems from its rapid degradation in body fluids, releasing active ions and recrystallizing into apatite under supersaturated conditions. This dynamic process is accompanied by continuous ion exchange: new ions are constantly incorporated into the lattice, while the degradation of apatite itself also releases ions, forming a cycle that maintains its excellent biocompatibility and activity (Jin et al., 2024). Studies have shown that Mg^2+^ and Zn^2+^ exert effects on the degradation of BG, ion release, and apatite formation, especially when present in high concentrations in the glass (Blochberger et al., 2015). Some studies suggest that the addition of magnesium ions inhibits the formation of apatite, and that this effect is more pronounced at higher magnesium concentrations (Al-Noaman et al., 2012). Zinc ions inhibit apatite formation by partially replacing calcium ions, thereby reducing the concentration of calcium ions released (Blochberger et al., 2015). After apatite formation, due to its greater thermodynamic stability, its degradation and ion release capacity is lower than that of BG. Therefore, controlling the timing of apatite formation is crucial to its active ion release efficiency. Based on this, it is worth investigating whether the co-doping of two ions can exert a synergistic effect to slowly inhibit apatite formation.

According to the current research on BG, ion doping is a very effective strategy for improving the performance of BG. However, previous studies have often focused on the biological effects of ions directly acting on cells or tissues, which typically exhibit significant concentration dependence, meaning that different ion concentrations can produce different or even diametrically opposed effects. The ion release concentration of BG is often determined by its chemical structure. The doping of multiple ions is not a simple additive process but rather forms a complex chemical system, which significantly influences the dissolution and recrystallization (mineralization) behavior of BG. This, in turn, alters the dynamic release of ions, ultimately affecting its biological performance. Therefore, this study incorporated magnesium and zinc, two commonly used functional ions, into BG. A series of characterization techniques confirmed the successful synthesis of the doped BG. The study then focused on whether different ratios of the two ions would affect the mineralization behavior and ion release concentration of BG, as well as their impact on *in vitro* osteogenic and angiogenic performance. It is hoped that this study will further refine the strategy of ion-doped modification of BG. The schematic workflow of this study is illustrated in Scheme 1.

## 2 Materials and methods

### 2.1 Preparation of BG

#### 2.1.1 Chemical reagents

Tetraethyl orthosilicate (TEOS), calcium nitrate tetrahydrate (Ca (NO_3_) _2_·4H_2_O), magnesium nitrate hexahydrate (Mg (NO_3_) _2_·6H_2_O), and zinc nitrate hexahydrate (Zn (NO_3_) _2_·6H_2_O) were obtained from Guangzhou Chemical Reagent Factory (China), along with absolute ethanol (EtOH). Additionally, hexadecyltrimethylammonium bromide (CTAB), triethanolamine (TEA), and cyclohexane were sourced from Aladdin (Shanghai, P.R. China). All chemicals were of analytical grade.

#### 2.1.2 Preparation of silica nanoparticle powders (SNP)

SNP (100% silica) were synthesized as mesoporous spherical particles via a sol-gel and two-phase layering approach (Xie et al., 2020). Briefly, 12 g of CTAB and 0.36 mL of TEA were dissolved in 108 mL of deionized water under gentle stirring at 60 °C for 1 h. Separately, 12 mL of TEOS was mixed with 48 mL of cyclohexane. The TEOS-cyclohexane solution was then slowly added to the aqueous phase and hydrolyzed at 60 °C for 8 h (150 rpm stirring). The resulting white precipitate was collected by centrifugation, washed three times with ethanol and deionized water to remove the template, and freeze-dried at 40 °C for 48 h.

#### 2.1.3 Preparation of different BG powders

BG powders with varying compositions were synthesized via solid-phase reactions, utilizing SNP as both a silicon source and a template. Specifically, 1.408 g of Ca(NO_3_)_2_·4H_2_O was dissolved in 40 mL of ethanol, followed by the addition of 1 g of SNP. The mixture was stirred for 12 h, corresponding to a composition of 60 mol% SiO_2_ and 40 mol% CaO. The suspension was stirred at room temperature until complete ethanol evaporation, yielding a precursor consisting of SNP loaded with Ca(NO_3_)_2_·4H_2_O. The precursor was then calcined at 600 °C for 5 h to obtain the final product, denoted as BG. This method was extended to prepare BG with different Si/Ca/Zn/Mg M ratios, and the samples were named according to their ionic compositions as listed in the Table 1.

**TABLE 1:** Abbreviations and the chemical composition of different sample groups.

Sample code	SiO_2_ mol.%	CaO mol.%	MgO mol.%	ZnO mol.%
BG	60	40	-	-
BG@Zn20/Mg0	60	20	20	-
BG@Zn15/Mg5	60	20	15	5
BG@Zn10/Mg10	60	20	10	10
BG@Zn5/Mg15	60	20	5	15
BG@Zn0/Mg20	60	20	-	20

### 2.2 Characterization of BG powders

#### 2.2.1 Phase characterization

Phase analysis of BG powders was conducted by employing an X-ray diffractometer (XRD, D-5000 Bruker AXS) with Cu Kα radiation (1.54178 Å). The angular range of 10°–70° (2θ) was analyzed.

#### 2.2.2 Chemical structure and composition analysis

The vibrational peaks of different groups of BG powders were determined using Fourier transform infrared spectroscopy (FTIR; VERTEX 70, Bruker, Germany) in the range of 400–4,000 cm^-1^; The Chemical composition of the BG powders was analyzed by X-ray photoelectron spectroscopy (XPS; K-Alpha, Thermo Fisher Scientific,USA), with C1s (284.8 eV) used as the standard for energy calibration of the XPS spectra.

#### 2.2.3 Microstructure

The surface morphology of BG was observed using a Merlin high-resolution field emission scanning electron microscope manufactured by Carl Zeiss in Germany (FE-SEM; Merlin, Carl Zeiss, Germany). Before observation, the powder was uniformly dispersed in ethanol, then dropped onto a copper plate and coated with platinum to ensure good conductivity of the sample.

### 2.3 Characterization of the mineralization behavior of BG powders

Different groups of bioactive glass powder were immersed in centrifuge tubes containing simulated body fluid (SBF) at a ratio of 10 mg/mL, and then placed in a shaking incubator for incubation at 37 °C and 60 r/min. After reaction times of 1, 3, 5, and 7 days, the samples were centrifuged to collect the supernatant, in which the ionic concentrations of Ca, Si, Zn, and Mg were measured. The reaction solutions corresponding to each time point were filtered through a membrane to obtain the mineralized BG powder. The collected powder was alternately washed three times with deionized water and ethanol, followed by drying in an oven at 60 °C. Finally, the powder was characterized for its phase composition and morphology using scanning electron microscopy (SEM) and X-ray diffraction (XRD), following the specific operational steps outlined in Section 2.2.

### 2.4 Co-culture of rBMSCs with BG powders

#### 2.4.1 Cell culture

Rat bone marrow mesenchymal stem cells (rBMSCs, ATCC, USA) were used to evaluate the cytological properties of the material. The cells were cultured in high-glucose DMEM (H-DMEM, Gibco, USA) supplemented with 10 vol% fetal bovine serum (FBS, Gibco, USA). BG powders were introduced into the culture plates to ensure uniform cell distribution. The cultures were maintained at 37 °C in a humidified atmosphere containing 5% CO_2_, with the medium refreshed every 2 days. For osteogenic differentiation, the medium was further supplemented with 10 nM dexamethasone, 50 μg/mL ascorbic acid, 10 mM β-glycerophosphate, and 50 μg/mL BG powders.

#### 2.4.2 Viability

In this study, rBMSCs were seeded in culture plates at a density of 2 × 10^4^ cells per well and co-cultured with BG powders for 1 day. Cell activity was assessed using a live/dead fluorescence kit (Calcein AM/EthD-III, Biotium, USA). After removing the culture medium, 150 μL of the prepared staining solution was added to each well in the dark, and the plates were incubated for 15 min at 37 °C. Stained cells were then observed and imaged using an inverted fluorescence microscope (ZEISS, Germany).

#### 2.4.3 Proliferation

The cell proliferation of rBMSCs cultured with various BG powders was assessed via the CCK-8 assay (Dojindo Laboratories, Japan). Briefly, cells were plated at 2 × 10^4^ cells/well and incubated with BG samples for 1, 3, and 5 days. After each time point, the culture medium was replaced with CCK-8 working solution, followed by 1 h incubation at 37 °C. Finally, the absorbance of the supernatant was measured at 450 nm wavelength using a microplate reader.

#### 2.4.4 ALP activity

rBMSCs were cultured in osteogenic differentiation medium for 7 days for ALP staining. The ALP staining was detected using BCIP/NBT Alkaline Phosphatase Color Development Kit (Beyotime, China).

#### 2.4.5 ARS (Alizarin red S) staining

rBMSCs were cultured in osteogenic differentiation medium for 14 days for ARS staining. The ARS staining was strained with ARS solution (Beyotime, China).

#### 2.4.6 Osteogenic differentiation

Osteogenic gene expression was assessed following the same culture protocol used for ALP activity. After 7 days of induction with BG powders, cells were gently rinsed three times with PBS and lysed. Total RNA was extracted using the HiPure Total RNA Micro Kit (Magen, China) and reverse transcribed into cDNA with the iScript cDNA Synthesis Kit (Bio-Rad, USA). Quantitative PCR was performed on an Applied Biosystems QuantStudio 6 Flex (ThermoFisher Scientific) to evaluate the expression of osteogenic markers, including *Runx2*, type I collagen (*Col-I*), and osteocalcin (*Ocn*). Relative gene expression levels were calculated using the 2^(–ΔCt) method, with GAPDH serving as the housekeeping gene.

### 2.5 Co-culture of HUVECs with BG powders

#### 2.5.1 Cell culture

Human umbilical vein endothelial cells (HUVECs) were purchased from Cyagen Biosciences (Guangzhou, China) and were cultured in endothelial cell medium (ECM, Cyagen, China) at 37 °C in a 5% CO_2_ atmosphere. The conditioned medium containing BG powders at a concentration of 50 μg/mL.

#### 2.5.2 Cell migration testing and analysis

The effects of the BG on HUVECs migration were evaluated using wound healing and Transwell assays. Prior to testing, cells were starved in low-serum (1%) medium for 24 h. For the wound healing assay, scratches were made with a 200 µL pipette tip, and debris was removed by PBS washing. After 12 h of culture in conditioned medium, cell migration was observed under an inverted microscope (Olympus, Japan), and the healing area was quantified using ImageJ. For the Transwell assay, HUVECs were seeded at 1 × 10^5^ cells/mL in the upper chamber (8 µm pore size, Corning, USA), while conditioned medium was added to the lower chamber. Following 12 h of incubation, cells on the lower membrane surface were fixed with 4% paraformaldehyde, stained with 0.1% crystal violet, and imaged. Migrated cells were counted using ImageJ.

#### 2.5.3 Tube formation assay

HUVECs were seeded onto Matrigel-coated 24-well plates at a density of 1 × 10^4^ cells/mL and cultured in the corresponding conditioned medium for 6 h. Tube formation was then observed and imaged using an inverted microscope (Olympus, Japan). The total tube length and number of junctions were quantified with ImageJ.

### 2.6 Statistical analysis

All results were reported as mean values accompanied by the standard deviation. Multiple group comparisons were conducted using one-way ANOVA analysis followed by a *post hoc* examination. Statistical significance was determined at *P < 0.05 or **P < 0.01 or ***P < 0.001.

## 3 Results and discussion

### 3.1 Phase, structure, and morphology of BG powders with different doping ions

**FIGURE 1:**
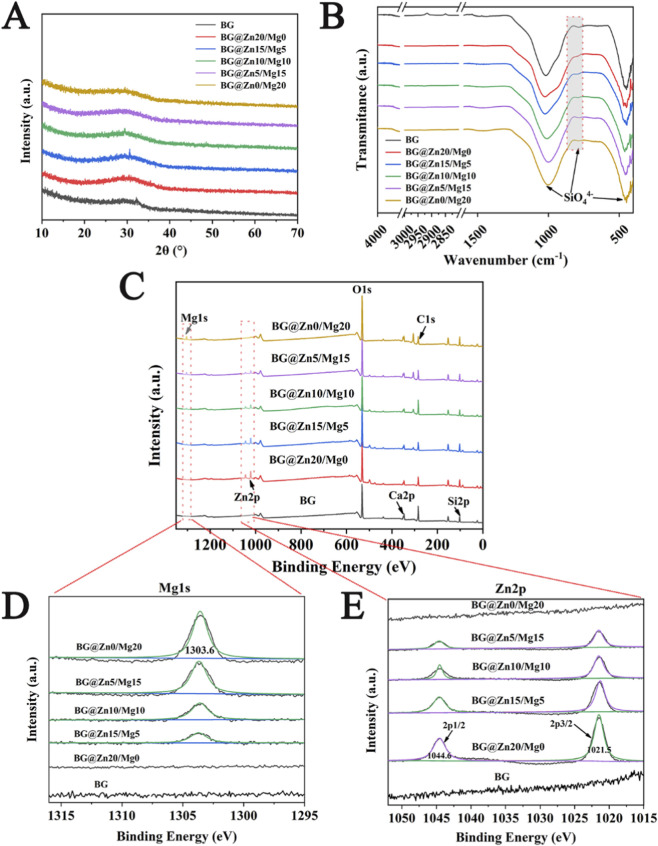
XRD patterns **(A)** FTIR spectra **(B)** XPS survey spectra **(C)** XPS high-resolution spectra of Mg1s **(D)** Zn2p **(E)** of the different BG powders.

Figure 1A shows the XRD diffraction pattern of the BG powders. As can be seen from the figure, the XRD patterns of the six different BG powder samples all exhibit a broad diffuse diffraction peak in the 2θ = 15°–30° range, which is a typical characteristic peak of an amorphous structure. This indicates that, compared to the initial BG, the BG powder samples doped with magnesium and zinc elements still exhibit a typical amorphous structure and have not crystallized due to the addition of these elements.

Figure 1B shows the FTIR spectra of BG powders with different ions doping. It can be seen that the infrared absorption spectra of different BG groups are basically similar, with obvious infrared absorption at 1,090 cm^-1^, 800 cm^-1^, and 475 cm^-1^, representing the asymmetric stretching vibration of Si-O-Si, the symmetric stretching vibration of Si-O-Si, and the symmetric bending vibration of Si-O-Si, respectively. With the doping of different elements, the positions of the characteristic absorption peaks show no significant shift, indicating that the appropriate addition of magnesium and zinc elements does not significantly affect the network structure of SiO_2_. However, the absorption peak at 800 cm^-1^ is weakened, indicating that the newly doped elements have entered the SiO_2_ network structure, disrupting the Si-O-Si bridge oxygen bonds in the glass network, leading to a decrease in the infrared absorption intensity of the Si-O-Si symmetric stretching vibration peak (Xie et al., 2020).

The XPS full spectrum (Figure 1C) shows that all BG samples contain Ca, Si, O, and C elements, and the doped Mg and Zn signals were successfully detected, with the intensity of their characteristic peaks (Mg1s, Zn2p) increasing with the doping amount. High-resolution spectra (Figures 1D,E) further confirm that the binding energies of the Mg 1s peak (1303.6 eV) and Zn 2p peak (1021.5/1044.6 eV) increase with rising doping concentration. Combined with XRD and FTIR results, this confirms the successful preparation of BG doped with different magnesium and zinc elements.

**FIGURE 2:**
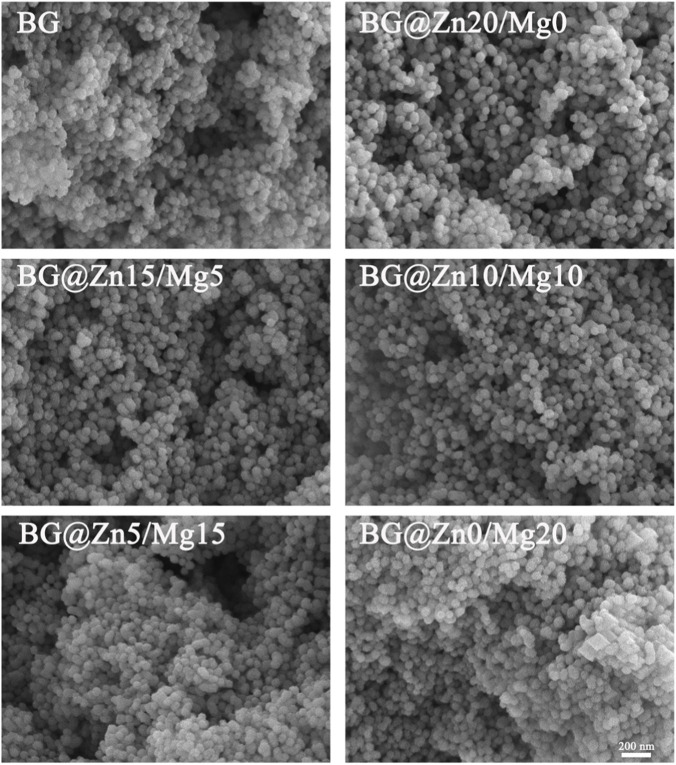
Scanning electron microscope (SEM) of the different BG powders.

To clarify the microstructure and size of the prepared BG powder, scanning electron microscopy (SEM) was used to observe the powder from different groups. As shown in Figure 2, the size of the prepared BG is approximately 50 nm, with a uniform size distribution and a predominantly spherical shape. Furthermore, the size and morphology remain largely unchanged with varying concentrations and ratios of magnesium and zinc elements. However, it is worth noting that as the magnesium doping concentration increases, spherical nanoparticles begin to sinter together. For example, this phenomenon has already begun to appear in the BG@Zn5/Mg15 group, and becomes more pronounced when magnesium doping reaches 20%. Relevant literature indicates that magnesium ions are functional elements that can promote bioactive glass sintering, and this phenomenon is also applicable to bioactive glass (Wetzel et al., 2020).

### 3.2 Mineralization behavior of different BG powders

Biodegradable BG affects cell behavior mainly by influencing the ionic composition of the surrounding medium, which indirectly mediates cellular responses (Lee et al., 2008; Schumacher et al., 2013). However, in this study, due to the low concentration of BG powder, its biological effect mainly depends on the release of active ions. Therefore, we need to focus on the relationship between the ion release level of BG after dual element doping and its cytological performance. Based on this, we verified whether the doping of two elements had any effect on the mineralization process and the corresponding changes in ion concentration through mineralization experiments with different BG groups.

**FIGURE 3:**
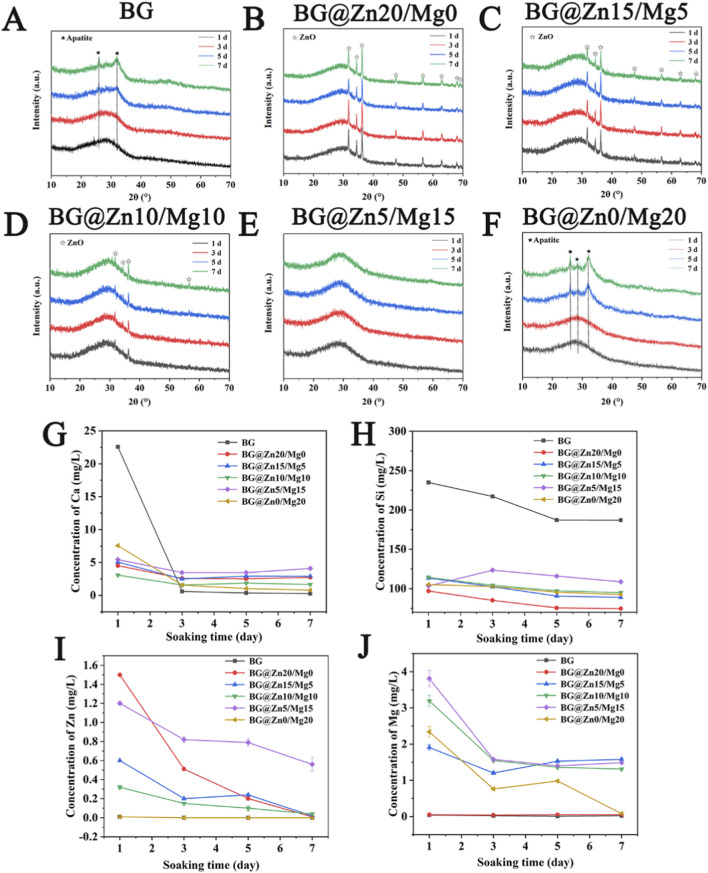
The XRD patterns of BG **(A)** BG@Zn20/Mg0 **(B)** BG@Zn15/Mg5 **(C)** BG@Zn10/Mg10 **(D)** BG@Zn5/Mg15 **(E)** BG@Zn0/Mg20 **(F)** after mineralization, and the corresponding Ca ion concentration **(G)** Si ion concentration **(H)** Zn ion concentration **(I)** and Mg ion concentration **(J)**.

Figures 3A–F show the differences in mineralization behavior among BG samples with different Mg/Zn doping ratios. The undoped sample (Figure 3A) exhibited weak HAp diffraction peaks (2θ = 26° and 32°) after 5 days of mineralization, while the high Zn-doped group (Figures 3B–D) showed ZnO characteristic peaks, suggesting that Zn^2+^ replacement of Ca^2+^ hindered mineralization and formed Zn(OH)_2_ dehydration products under alkaline conditions. The intensity of the ZnO peak decreases with decreasing Zn content and increasing mineralization time (thermodynamically unstable). When the Zn/Mg co-doping ratio is 5%/15% (Figure 3E), mineralization is significantly inhibited; however, the pure Mg-doped group (Figure 3F) still forms distinct HA diffraction peaks.

Figures 3G–J shows the differences in ion release behavior during mineralization. In the undoped BG group (Figures 3G,H), the initial release of Ca^2+^ and Si^4+^ was the highest, followed by a rapid decrease in Ca^2+^ concentration (due to the consumption of HAp crystals), while Si^4+^ decreased more slowly. The Zn-doped group (Figure 3I) exhibited lower Zn^2+^ concentrations (presumably due to the formation of Zn (OH)_2_ precipitates), with Ca^2+^ release inhibited, but Si^4+^ concentrations remained at ∼100 ppm. Notably, the Mg/Zn co-doped group (15%/5%) exhibited the slowest decline in ion concentrations (Figure 3J), indicating synergistic inhibition of mineralization crystallization by both ions; the pure Mg-doped group showed no such effect.

**FIGURE 4:**
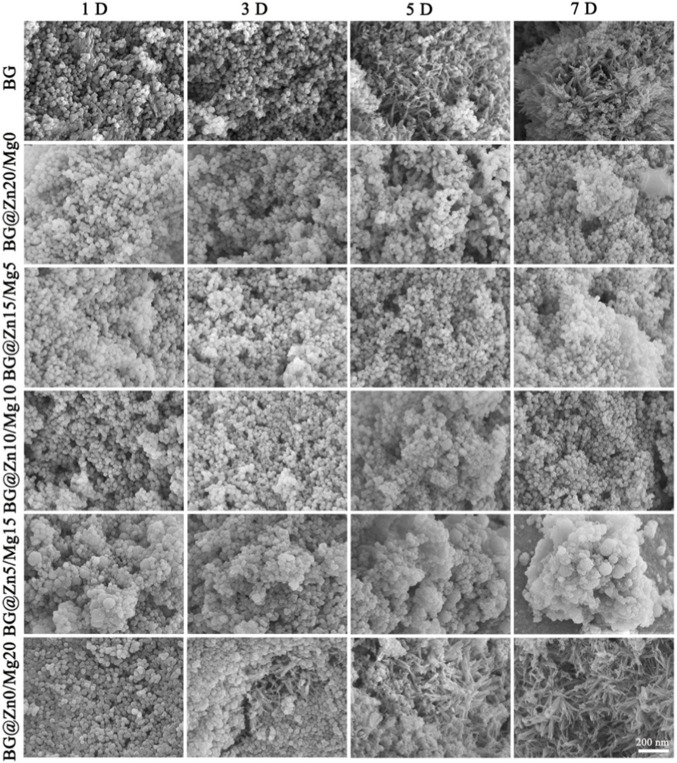
The SEM images of BG, BG@Zn20/Mg0, BG@Zn15/Mg5, BG@Zn10/Mg10, BG@Zn5/Mg15, BG@Zn0/Mg20, after mineralization for different days.

Through systematic observation and analysis of the microscopic morphology of bioactive glass (BG) powders from different groups during the mineralization process (as shown in Figure 4), it was found that their mineralization behavior is significantly influenced by the type and concentration of doped elements. The undoped group exhibited a transition from regular spherical shapes to irregular geometric shapes as early as the initial stage of mineralization (1 day), consistent with ion release kinetic data, indicating that the material had entered a rapid degradation phase. However, no new phases were detected during this stage. As mineralization progressed to 3–5 days, nanoscale secondary particles appeared on the particle surfaces and in the interstitial regions, suggesting that nucleation processes may have occurred in the amorphous phase. In the late mineralization stage (5–7 days), characteristic needle-like apatite crystals formed.

The high-concentration Zn-doped group (10%–20% Zn) exhibited unique mineralization kinetic behavior. The initial degradation morphology was similar to that of the control group, but irregular micron-sized particles formed during the mid-mineralization phase (three to five days), which were speculated to be a zinc hydroxide passivation layer that hindered Ca ion release and thus inhibited apatite formation.

The Zn/Mg co-doped group (5%Zn+15%Mg) exhibited a significantly different amorphous phase mineralization pathway, with rapid surface etching occurring within 1 day of mineralization, accompanied by the formation of larger irregular plate-like structures. Combined with XRD amorphous diffraction peaks (2θ = 20°–35°), this was confirmed to be the amorphous calcium phosphate (ACP) phase. This morphological evolution difference suggests a synergistic effect between Mg^2+^ and Zn^2+^ in influencing crystallization kinetics. In contrast, the mineralization trajectory of the single Mg-doped group (15% Mg) was consistent with the control group, indicating that Mg^2+^ may promote heterogeneous nucleation of HAp through surface energy regulation but does not alter the fundamental mineralization mechanism.

Studies have shown that ACP is widely present in the body and exhibits characteristics like BG in terms of atomic arrangement. For example, both are unstable and prone to transforming into crystalline calcium phosphate (Pei et al., 2018). Certain trace elements in the body (magnesium and zinc) adsorb onto the surface of ACP or replace calcium ions to disrupt the crystallization process and stabilize ACP. The presence of a specific amount of ACP may be key to bone tissue mineralization and high activity (Lowenstam and Weiner, 1985). Therefore, this study adopted a dual ion doping strategy to maintain the amorphous state of the BG. Based on the XRD and SEM results after mineralization, it was found that replacing calcium ions with high concentrations of zinc ions significantly inhibited the mineralization of BG, while doping BG with high concentrations of magnesium ions did not hinder mineralization. Similar studies have also revealed that replacing calcium ions with low concentrations of zinc ions did not result in the formation of hydroxyapatite (Neščáková et al., 2019). However, there are conflicting views on the effect of magnesium doping on mineralization. Some studies claim that magnesium can promote the formation of apatite, but there are also studies that show the opposite results (Vallet-Regí et al., 1999; Oliveira et al., 2002). In this study, it was found that the co-doping of the two elements, with a ratio of Zn5%/Mg15%, significantly inhibited the formation of apatite and formed a substance like ACP, with relatively stable corresponding ion concentrations. This may be a key factor affecting its cytological performance.

In summary, all groups follow the dissolution-reprecipitation mineralization mechanism, but the doped elements influence this process through different pathways: the promoting effect of Mg^2+^ may be related to reducing the activation energy for the ACP-to-HAp phase transition; while the unique behavior of the co-doped group suggests the existence of a Mg-Zn synergistic effect. These findings provide important insights for the composition design of bioactive glasses, enabling programmable control of mineralization kinetics through precise regulation of the Zn/Mg ratio, which holds significant implications for achieving time-dependent functional properties in bone repair materials.

### 3.3 Cytological properties of different BG powders

Due to the high level of active ion release from BG, it may often be detrimental to cell growth. Therefore, it is first necessary to determine the appropriate BG concentration suitable for cell growth.

**FIGURE 5:**
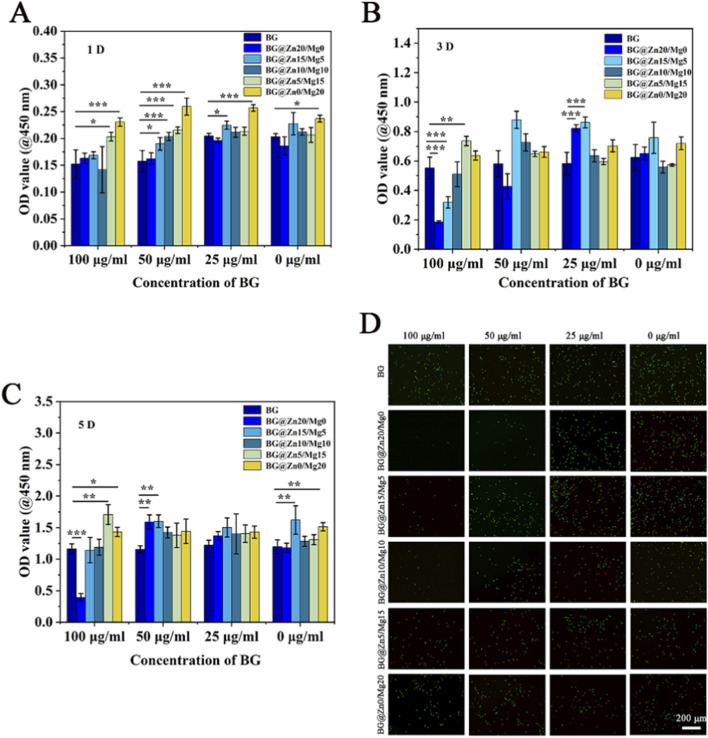
Proliferation of rBMSCs after co-culturing for 1D **(A)** 3D **(B)** 5D **(C)** and Live/dead staining fluorescence images **(D)** with different BG powders. n = 4, *p < 0.05, **p < 0.01, ***p < 0.001.

Cell proliferation was first assessed using the CCK-8 assay. At a culture time of 1 d, magnesium ions were found to significantly promote cell proliferation (as shown in Figure 5A). As the culture time increased, the overall number of cells continued to rise. However, different concentrations and ion compositions of bioactive glass significantly influence cell proliferation. BG with high zinc ion content exhibited cytotoxicity at high concentrations, but as the concentration decreased, the toxicity also decreased. Additionally, bioactive glass with dual ions demonstrated better cell proliferation-promoting capabilities (as shown in Figures 5B,C). From the cell viability assay, it can also be observed that the group with a high Zn ion ratio exhibited significant toxicity at high concentrations, with poor cell spreading and fewer cells, which was consistent with the results of the proliferation assay (as shown in Figure 5D).

**FIGURE 6:**
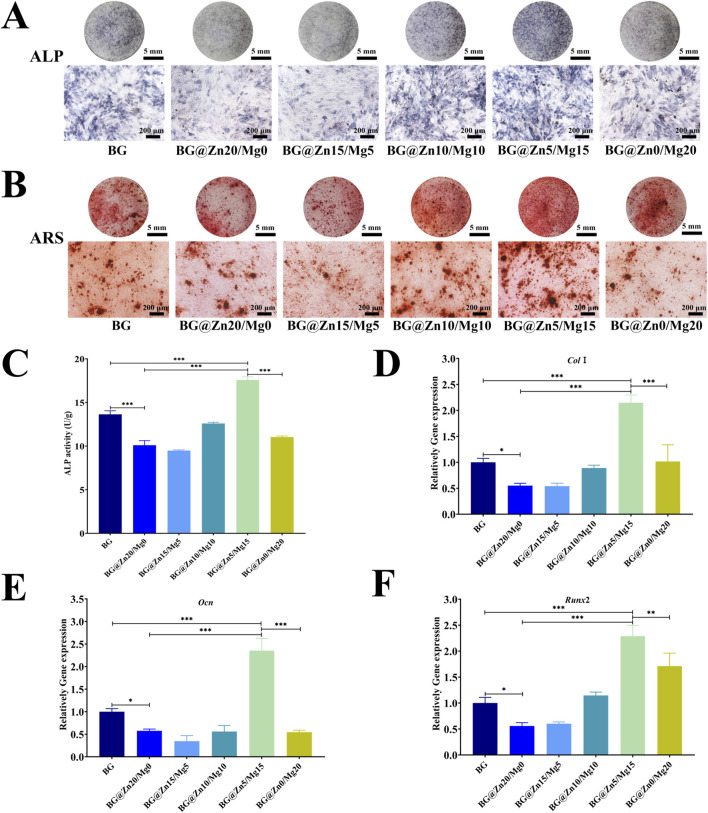
ALP staining images **(A)** and quantitative ALP activity at day 7D **(C)**. Alizarin red staining images at day 14D of BMSCs after being treated with different BG powders **(B)**. The statistical data of osteoblast-related genes including *Col1* **(D)** *Ocn* **(E)** and *Runx2* **(F)** after BMSCs being treated with different BG powders. n = 4, *p < 0.05, **p < 0.01, ***p < 0.001.

The staining and quantitative analysis results of ALP indicate that BG materials with different magnesium-zinc doping ratios exert a significant regulatory effect on the early osteogenic differentiation of rBMSCs. The undoped BG group exhibited basal ALP activity, while the introduction of zinc significantly inhibited ALP expression. In contrast, the incorporation of magnesium exhibited a dose-dependent promotional effect, with the BG@Zn5/Mg15 group demonstrating the most optimal osteogenic effect. Notably, when the magnesium doping ratio exceeded 15%, ALP activity decreased, indicating that magnesium and zinc ions have an optimal synergistic ratio (15% Mg/5% Zn) for regulating osteogenic differentiation. Both excessively high or low doping ratios can impair osteogenic promotion effects (as shown in Figures 6A,C).

Mineralization nodule analysis (Figure 6B) revealed that BG with different magnesium-zinc doping ratios significantly influenced the calcium salt deposition capacity of rBMSCs. The undoped BG group exhibited obvious calcium nodule formation, confirming the basal osteogenic activity of BG. The introduction of zinc reduced calcium nodule deposition, while magnesium incorporation reversed this inhibitory effect, achieving optimal results at a 15% Mg/5% Zn ratio. Notably, when the magnesium doping level increased to 20%, the osteogenic effect decreased, further confirming that magnesium and zinc ions exhibit optimal synergistic osteogenic activity at a 15%/5% ratio.

Analysis of osteogenic-related gene further confirmed the critical influence of the magnesium-zinc doping ratio on the osteogenic-promoting capacity of BG. Experimental measurements of the expression levels of typical osteogenic markers such as *Ocn, Col1, and Runx2* (Figures 6D–F) revealed that the undoped BG group exhibited a certain degree of basal osteogenic activity, while high zinc doping significantly inhibited the expression of these osteogenic markers. With the introduction of magnesium, osteogenic-related indicators showed a gradual upward trend, with the 15% Mg/5% Zn co-doped group exhibiting the most optimal osteogenic promotion effect. This finding is consistent with previous results from ALP activity and mineralization nodule experiments, collectively indicating that magnesium and zinc elements can produce the optimal synergistic effect at a specific ratio (15%/5%), maximizing the osteogenic induction capacity of BG materials.

In this study, high concentrations of Zn doping are detrimental to cell proliferation and subsequent osteogenic differentiation. As shown in Figure 5, the safe concentration of Mg is higher than that of Zn. Research indicates that zinc ion concentrations exceeding 0.09 mM (5.85 ppm) begin to inhibit the normal function of osteoblasts, and concentrations exceeding 0.150 mM (9.75 ppm) can lead to programmed cell death. (Yamamoto et al., 1998; Wätjen et al., 2002). Magnesium ions have been shown to promote cell proliferation and differentiation at higher concentrations. Studies by Nourisa et al. (Nourisa et al., 2021) and Zamani et al. (Zamani et al., 2019) have both confirmed the promotional effect of magnesium ions on osteoblast activity, the former reported that magnesium ion concentrations of 2–10 mM significantly promote cell proliferation and differentiation, while the latter found that concentrations of 3–6 mM have the most significant enhancing effect on osteoblast proliferation.

From the above studies, it can also be concluded that the excessive zinc doping in this study may have exceeded the optimal concentration range suitable for cellular metabolic activities. Therefore, as the magnesium/zinc doping ratio increases, the osteogenic potential of the cells also improves, as shown in Figure 5. However, a higher magnesium doping ratio is not necessarily better. When the zinc doping ratio decreases to 5%, the synergistic effect between zinc and magnesium maximizes the promotion of osteogenic differentiation in rBMSCs.

**FIGURE 7:**
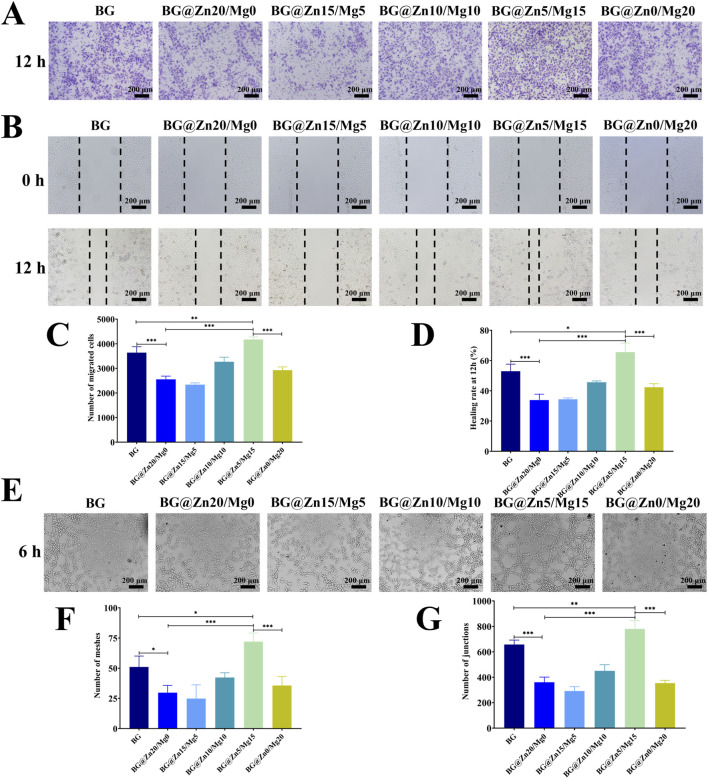
The transwell test showing the ability of HUVECs to migrate after co-culturing for 12 h **(A)**, scratch migration assay **(B)** and quantitative analysis **(C,D)** of HUVECs after co-culturing for 12 h. The representative images of the tube formation assay **(E)** and quantitative analysis **(F,G)**. n = 4, *p < 0.05, **p < 0.01, ***p < 0.001.

To compare the angiogenic activity of different magnesium and zinc-doped bioactive glass *in vitro*, HUVECs were cultured in media containing various bioactive glass, and the migration and angiogenic capabilities of HUVECs were systematically investigated. The migration assays revealed that BG@Zn5/Mg15 significantly enhanced the migration of HUVECs, with a notably higher number of migrated cells observed after 12 h compared to other groups *in vitro* (Figures 7A,C). Consistent with the migration assay results, the scratch wound healing experiments demonstrated that the BG@Zn5/Mg15 group exhibited the highest wound healing rate (Figure 7B), achieving 65% closure after 12 h (Figure 7D). These indicated that the Mg^2+^ and Zn^2+^ doping ratio in BG@Zn5/Mg15 possessed a strong ability to promote HUVECs migration.

To further explore the angiogenic potential of different magnesium and zinc-doped BG, HUVECs were cultured on a Matrigel surface to induce tube formation. The results showed that the tube formation in the BG@Zn5/Mg15 group was characterized by the most complete structures, the highest number of meshes and nodes (Figures 7E–G). The above results suggested that BG@Zn5/Mg15 exhibited a strong capacity to enhance HUVECs migration and angiogenesis, providing a favorable reference for subsequent HUVECs studies aimed at promoting vascularization.

Magnesium and zinc have both been proven to promote vascularization (Lai et al., 2019; Xu et al., 2019; Lu et al., 2021). Therefore, this study continues to maximize the pro-vascular effects of double ions through a double ion doping strategy. Studies have shown that Zn ion concentration at 3.6 ppm can achieve optimal angiogenesis effects, while Mg ion concentration at 120 ppm can achieve optimal angiogenesis. It has also been found that angiogenesis promotes osteogenesis (Liu et al., 2020; Yu et al., 2023). In addition, zinc ions and magnesium ions were found to be beneficial to the migration and maintenance of endothelium, which is consistent with the conclusions of this study (Figures 7A–D) (Wang et al., 2016; Chen et al., 2018). Although the ion concentration released by BG in this study was lower than this concentration, it is not difficult to infer from the results of previous cytological experiments that the actual local release concentration should be much higher than 3.6 ppm. This is also the reason why the angiogenesis -related indicators decreased when the Zn element doping concentration was too high. However, after doping with the safer magnesium ion, angiogenesis was maximally promoted, proving the synergistic promotion of angiogenesis by both elements in HUVECs.

## 4 Conclusion

This study demonstrates that co-doping Mg^2+^ and Zn^2+^ into BG at optimized ratios effectively modulates its mineralization behavior and enhances osteogenic and angiogenic properties. Specifically, the 5%Zn/15%Mg doping ratio exhibited the most favorable performance by maintaining the amorphous state of BG, thereby inhibiting rapid hydroxyapatite formation and sustaining higher ion release concentrations, which can enhance osteogenic activity, as evidenced by improved rBMSCs response and promote angiogenesis, supported by superior HUVECs performance. These findings reveal that multi-ion co-doping induces a synergistic effect, allowing precise control over BG’s mineralization kinetics and biological functionality. The optimized BG@Zn5/Mg15 composition established in this study exhibits significant potential for clinical translation, particularly for treating critical-size bone defects and osteoporotic fractures where delayed healing and poor vascularization are major challenges. The sustained ion release profile and synergistic osteo/angiogenic effects make this material suitable for applications requiring prolonged bioactivity, such as spinal fusion, maxillofacial reconstruction, and fracture non-unions.

## Data Availability

The datasets presented in this study can be found in online repositories. The names of the repository/repositories and accession number(s) can be found in the article/Supplementary Material.
