# Supplementary material for: Optimizing the osteogenic and angiogenic properties of nano-bioactive glass through adjustment of zinc and magnesium ion doping ratios: an in vitro study
Source: Front Bioeng Biotechnol. 2026 Apr 24;14:1831550. doi: 10.3389/fbioe.2026.1831550 (PMC13153095; doi:10.3389/fbioe.2026.1831550)
Supplement: Supplementary file 1 [file Table1.docx]

Table S1 Primer sequences used for RT-PCR in the study.

| Gene | Direction | Sequence (5’-3’) |
| --- | --- | --- |
| GAPDH  Col Ⅰ  OCN  Runx2 | Forward  Reverse  Forward  Reverse  Forward  Reverse  Forward  Reverse | TGTGTCCGTCGTGGATCTGA  TTGCTGTTGAAGTCGCAGGAG  ATGCCGCCACCTCAAGATG TGAGGCACAGACGGCTGAGTA  AGCAGCTTGGCCCAGACCTA  TAGCGCCGGAGTCTGTTCACTAC  CACTGGCGGTGCAACAAGA  TTTCATAACAGCGGAGGCATTTC |
